# Annotations

**Published:** 1900-12-01

**Authors:** 


### ANNOTATIONS.

### French Medical Men on Sanatoria.

A lively discussion, which is at present going on in France regarding the merits and demerits of treating tuberculosis in sanatoria, is recorded in the columns of the *Journal de Médecine de Paris*. Dr. Guelpa, who upholds the sanatorium treatment, protests against the statements of Drs. Dignat and Bardet, who do not incline to it. The first-mentioned complains that his opponents pretend that his statement that consumptives are best treated in special institutions implies that they cannot be cured elsewhere. Of course they can be successfully treated at home if their friends have the means, the knowledge, and, above all, the control over the patient which the doctor in the sanatorium has. Dr. Guelpa protests that he does not claim more for a consumption hospital than people do for any other; that the patient is properly treated there, whether he will or no; and that he is prevented from infecting those who come in contact with him. A secondary advantage is the spreading of hygienic knowledge by the precautions taken by ex-patients when they come back to their homes. Dr. Dignat, on the other hand, regards the placing of consumptives in a sanatorium as being equivalent to the segregation to which lepers are subjected. Dr. Bardet seems to think that the sanatorium must always be a luxury for the rich, who can afford to go far afield in search of health into those climates which long custom has associated with the relief of consumption. But on this point Dr. Guelpa protests that what is wanted is rather a treatment than a climate, and that equally good results have been obtained at Davos, among the snow-clad hills, on the sea coast, and at a number of places at varying heights between. Another objector, Dr. de Cresantigues, complains that consumptives, classed as cured at a sanatorium, often develop their malady again when they return home. To this Dr. Guelpa retorts: "Does Dr. de Cresantigues not consider that he has cured a case of rheumatism because afterwards the patient has another attack?" On the whole, the champion of the sanatorium seems to hold his own.

### The Training of the Officer.

All the schemes of Army reform, of which so many are in the air, deal with some improvement in the condition of the private soldier, and with devising means to enable him to live as an officer, if by his courage he wins the chance of a commission. But to the end of time the majority of officers will probably be drawn from another class than the rank and file, even though they are compelled to begin their career at the foot of the tree. And no scheme seems to have been thought of for improving the officer. He has, indeed, to go through a grind before he enters the Army, but it is by no means certain that this grind is the best preparation for his life-work. The Army crammer is a recognised institution, and cram prepares for nothing but examinations. A youth who hopes to enter the Army must spend long hours a day for months indoors, studying subjects which, in nine cases out of ten, he will never look at after he gets through his examination. Is this the best preparation for a life that will probably involve a good deal of hardship and exposure to the elements, and, if he is to be worth more to his country than one of the men under his command, a quick observation of the features of the country he is in, of the tracks of animals and men, of all those details by means of which one foe outwits another? This is the knowledge which savages have, and which is admittedly lost by civilised man. The bookworm, which the Army student must needs for a time be, loses, in proportion to his concentration on books, just those faculties which men used to an open-air life, to sport, to danger—whether they be savages or civilised—possess and develop. This point is well brought out in an article by Dr. H. H. Almond, head-master of Loretto School, which appears in the October number of the *Nineteenth Century*. Dr. Almond protests that the Army student is not educated for his life-work. He quotes a statement of the head-master of Dulwich, that "every master of an Army class knows that the moment a boy joins that class his education stops and his cramming begins." And, moreover, he holds that the boy who would be most useful in the Army will not submit to undergo the training for the examination. He quotes a case which could no doubt be paralleled by many others. The head boy of a certain house in a certain large school was considered by his house-master to be "a splendid fellow." He had put down bad language in the house, he permitted no bullying; he made the loafers go out on wet afternoons, instead of brewing strong tea and eating pastry in their over-heated studies. That was one point of view. But another master considered this same boy a failure. "Do you know," he said, "that if that boy had worked, he could have got into the Army?" But this healthy, wholesome boy, this born leader of his fellows, would not do the "work" required for the Army examination. The career for which Nature seemed to have fitted him was not to be his, and any one of the loafers whom he drove out in the rain to gain health and strength had a better chance of gaining a commission than he. It is possible that there is something to be said on the other side, and that a good deal of scientific knowledge is needed for the successful soldier; but it is noteworthy that more than one of our most popular and successful officers have either failed to pass or refused to go in for the Army examinations, and have made their way into the profession by way of the Militia.

### Homœopathy and Malaria.

It seems that a meeting was held recently, under the auspices of the African Trade Section of the Liverpool Chamber of Commerce, at which many ladies and others were gathered together, to listen to an account of the new views on the cause, prevention, and treatment of malarial fever, the whole matter being treated from a "homœopathic" standpoint. The matter would be of no particular interest were it not that the outcome of the discourse was a statement by the chairman of the meeting that if the lecturer would bring him a qualified homœopathic practitioner he would be very glad to get him an appointment on the African Coast. Now it may fairly be hoped that no such nonsense will be indulged in; but if any such foolish thing should be done as sending a "homœopathist" to play with the lives of people in malarious countries, we hope that some care will be taken to ascertain and to make public the exact means taken by him in the treatment of the disease. It is necessary to pin homœopathists down to their creed, and not to let them escape either from the doctrine of similars or from infinitesimal doses. The preposterous claims so often put forward on their behalf make it especially desirable that no hocus pocus should be permitted in regard to this malaria question, and if homœopathists use quinine in the treatment of this disease they ought to show that the drug is administered on homœopathic principles and in homœopathic doses. If in curing a malarial fever these gentlemen find it necessary to use quinine in ordinary doses, their claims fall to the ground.

